# A very rare localization of a rare disease: palmar lichen nitidus^☆^

**DOI:** 10.1016/j.abd.2021.03.009

**Published:** 2021-11-24

**Authors:** İrem Nur Durusu, Dilara Güler, Gülhan Gürel, Gülsüm Şeyma Yalçın

**Affiliations:** a Department of Dermatology, Afyonkarahisar Health Sciences University, Afyonkarahisar, Turkey; b Department of Pathology, Afyonkarahisar Health Sciences University, Afyonkarahisar, Turkey

**Keywords:** Dermoscopy, Isolated, Lichen nitidus, Palmar, Palmoplantar

## Abstract

Lichen nitidus is an uncommon lichenoid dermatosis that could be defined as multiple, separated, shiny, pinpoint, pale to skin-colored papules. Palmoplantar lichen nitidus is a quite rare variant of lichen nitidus. It is hard to make a diagnosis of palmar lichen nitidus when there are no lesions elsewhere on the body. There are some dermoscopic features defined for both palmoplantar and non-palmoplantar lichen nitidus that might be useful to facilitate the diagnosis before histopathological examination. Herein, we report a case of a 24-year-old man diagnosed with isolated palmar lichen nitidus with dermoscopic features and histopathological confirmation.

## Case report

**Figure 1:**
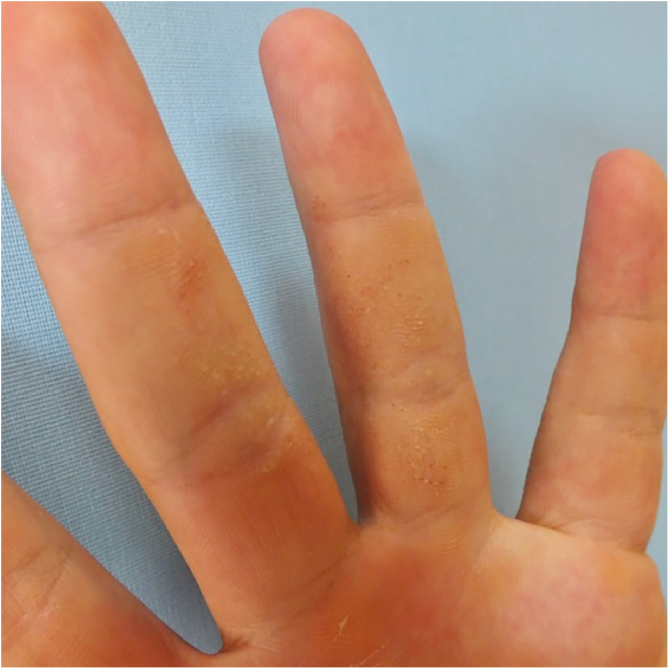
Asymptomatic, pitted, hyperkeratotic, grouped papules on the palmar aspect of the third and fourth phalanges of the left hand of a 24-year-old man.

**Figure 2:**
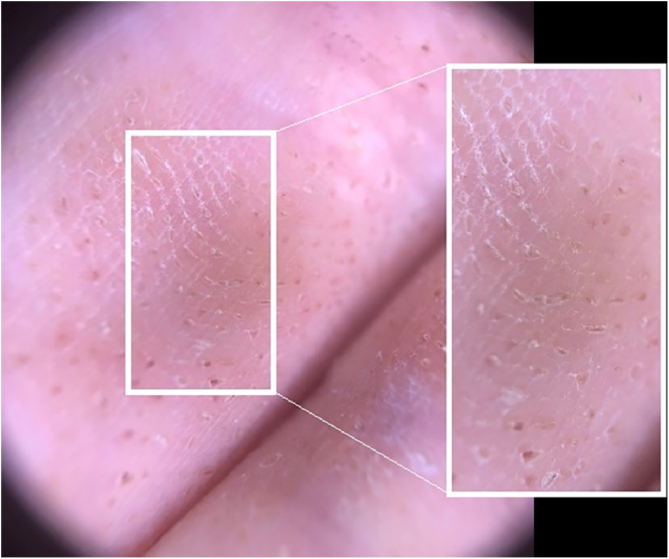
Fine round-to-ovoid comedo-like central depression areas surrounded by white halo-like scales and white linear scales connecting the whole structure.

**Figure 3:**
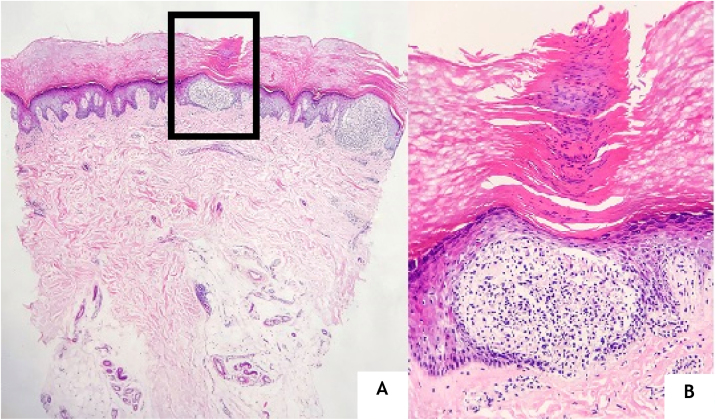
(A), Two “ball and claw” appearances in the middle and right side of the photo (Hematoxylin & eosin, ×40). (B), Epidermal collarette surrounding the dermal lymphohistiocytic infiltrate and a parakeratotic focus located right above it (Hematoxylin & eosin, ×200).

A 24-year-old man presented to us with asymptomatic, pitted, hyperkeratotic, grouped papules on his third and fourth phalanges at the palmar aspect of left hand with a duration of four years (Fig. 1). His past medical history and family history were not significant. A whole-body examination including oral and genital mucosa was normal. The differential diagnosis of nevus comedonicus and lichen nitidus (LN) was established. With dermoscopic examination (DermLite DL4; 3 Gen; polarized, 10×) fine round to ovoid comedo-like central depression areas surrounded by white halo-like scales and white linear scales connecting the whole structure were detected (Fig. 2). A 3 mm punch biopsy was performed. The histopathology revealed a few parakeratotic foci and hyperkeratosis in other surfaces. There were well-circumscribed lymphohistiocytic infiltrate in the papillary dermis expanding a small number of dermal papillae. Epidermal collarettes, elongated claw-like rete ridges wrapped around the subepidermal infiltrates (Fig. 3a). There were occasional multinucleated giant cells within the lymphohistiocytic infiltrates. The parakeratotic foci were located right above the “ball-and-claw” areas (Fig. 3b). There were minimal perivascular lymphocytes around the superficial dermal vascular plexus. The diagnosis of palmar LN was established with current clinical, dermoscopic, and histopathologic findings. The patient was given topical keratolytic, corticosteroid, and emollient treatment and followed up. The lesions have improved after treatment based on his teledermatological assessment performed two months later.

The patient was evaluated in the dermatology clinic of Afyonkarahisar Health Sciences University Medical Faculty.

## Discussion

LN is an uncommon lichenoid dermatosis that could be defined as multiple, separated, shiny, pinpoint, pale to skin-colored papules, 1 to 2 mm in diameter. LN usually affects children and young adults without any sex predilection. The etiology of the disease is uncertain.^1^ The most commonly involved sites are flexor areas of upper extremities, hand dorsums, trunk, and genitalia.^2^ LN located solely at palms presenting as hyperkeratotic pitted papules and plaques have been rarely reported.^3^ It is hard to make a diagnosis of palmar LN when there are no lesions elsewhere on the body. There are some dermoscopic features defined for both palmoplantar and non-palmoplantar LN that might be useful to facilitate the diagnosis before histopathological examination. Herein, we report a case of a 24-year-old man with isolated palmar LN to contribute to the literature and emphasize the diagnostic importance of the dermoscopic description for such a very rare disease.

Isolated palmar LN is a quite rare disease that presents with asymptomatic or occasionally pruritic hyperkeratotic pitted papules. Park et al. reported that palmar involvement usually seemed to accompany LN lesions of typical sites and claimed that there have been only 4 cases of LN confined to the palm including their case.^4^ Similarly, Podder et al reported a case of isolated palmar LN and claimed that this was the first case from Eastern India.^3^

There are a few dermoscopic findings defined for LN in the literature. These definitions are quite different between the palmoplantar variant and classical LN. The absence of dermatoglyphics, radial ridges, central depression areas with non-polarised mode; ill-defined hypopigmentation with diffuse erythema, and linear vessels with polarised mode were observed in classical LN.^5^ Malakar et al. indicated that dermoscopy of forearm demonstrates multiple, white, well-circumscribed, circular areas and an indistinct brown shadow reflected through these white circles.^6^ In the palmoplantar variant specifically; linear parallel scales discontinued by oval, well-defined depressions and surrounded by ring-shaped, silvery-white scales were described.^7^ In the case here reported ovoid, comedo-like central depressions surrounded by white halo-like scales and communicating white linear thicker scales parallel to the long axis of ovoid structures were observed.

The histopathological main features of LN are well-circumscribed infiltration of lymphocytes, macrophages, Langerhans giant cells, and multinucleated epithelioid histiocytes in the papillary dermis surrounded by the elongated epidermal rete ridges.^1^, ^2^ This subepidermally located lymphohistiocytic infiltrate surrounded by acanthosis, the parakeratotic epidermis is the so-called “ball-in-clutch” or “ball-in-claw” appearance.^5^ There were similar histopathological findings in the case here reported.

The presence of white halo-like, well-demarcated structures probably correspond to epidermal acanthosis on histopathology while the brown shadow-like central depressions are considered a reflection of the underlying dense inflammatory infiltrate composed of lymphocytes and epitheloid cells.^6^

Many treatment modalities have been tried including topical and systemic corticosteroids, antihistamines, acitretin, UVA, narrowband UVB, and cyclosporin for LN.^8^ Most lesions were asymptomatic and limited to a small part of the body not requiring intensive treatment with serious side effects on the other hand. We have observed some improvement of the lesions under treatment with topical corticosteroids and keratolytic agents.

Isolated palmar LN is a very rare disease most commonly affecting young patients. Dermoscopy is a noninvasive, cheap, and fast method to diagnose the disease and might be used to make the diagnosis and reduce the need for biopsy.

This case report was presented as an oral presentation in the International Dermatology, Dermatopathology and Esthetics Academy 2020 Congress.

## Financial support

None declared.

## Authors' contributions

İrem Nur Durusu: Approval of the final version of the manuscript; critical literature review; data collection, analysis, and interpretation; effective participation in research orientation; ıntellectual participation in propaedeutic and/or therapeutic; management of studied cases; preparation and writing of the manuscript; study conception and planning.

Dilara Güler: Approval of the final version of the manuscript; critical literature review; data collection, analysis, and interpretation; effective participation in research orientation; ıntellectual participation in propaedeutic and/or therapeutic; management of studied cases, study conception, and planning.

Gülhan Gürel: Approval of the final version of the manuscript; critical literature review; effective participation in research orientation; ıntellectual participation in propaedeutic and/or therapeutic; management of studied cases; manuscript critical review; study conception and planning

Gülsüm Şeyma Yalçın: Approval of the final version of the manuscript; critical literature review; effective participation in research orientation; ıntellectual participation in propaedeutic and/or therapeutic; management of studied cases; study conception and planning.

## Conflicts of interest

None declared.
